# P271: Knowledge of infection prevention and control during pregnancy in local health facilities in Bujumbura

**DOI:** 10.1186/2047-2994-2-S1-P271

**Published:** 2013-06-20

**Authors:** R Paul-Claudel

**Affiliations:** 1 Directeur de l'Offre et de la demande de soins, Bujumbura, Burundi

## Introduction

Nosocomial infection is a constant concern in the obstetric and surgical practice in both developing countries than in developed countries.

## Objectives

To assess knowledge, attitudes and practices of the staff of maternity services in the prevention of nosocomial infection in three community health centers in Bujumbura.

## Methods

A qualitative survey was conducted using a standardized questionnaire over 2 months with 76 health workers from three maternity wards of health facilities in the vicinity of all categories. Data collection consisted of two parts: first assessment of knowledge through a questionnaire and also the observation of attitudes and practices of personal hygiene.

## Results

It was noted: In knowledge: Knowledge of the exact def-inition of nosocomial infection: 75% (doctors), modes of transmission of infection (manu-portage): 50%, hygiene protocols: 25%, and procedures decontamination of contaminated equipment: 16%. At the observation of practices:

Compliance with hand washing before and after childbirth: 50% Use of alcohol-based solutions: 20%, antiquated equipment sterilization: 80% Compliance with the sterilization of equipment: 25% Circulation in delivery rooms disorganized: 100%, perceived risk of blood contamination (uterus): 75%, biomedical waste collected and decontaminated evil: 80%, existence of incinerators: 33.3% and participation in training in personal hygiene: 50%.

## Conclusion

There is a high risk of infectious contamination in maternity services studied for both the staff and for parturient and their newborns.

## Competing interests

None declared.

